# FACTORS ASSOCIATED WITH HEALTH-RELATED QUALITY OF LIFE IN KOREAN ELDERLY BY REGION

**DOI:** 10.1093/geroni/igz038.3410

**Published:** 2019-11-08

**Authors:** Jungmi Yun, Yeongsuk Lee, Hyun-Ju Lee

**Affiliations:** 1 Graduate School, College of Nursing, Pusan National University, Busan, Korea, Republic of; 2 College of Nursing, Catholic University of Pusan, Busan, Korea, Republic of

## Abstract

There have been inequalities in healthcare indices in the elderly by regions in Korea, and this gap is increasing. To improve their health-related quality of life (HRQoL), it is essential to identify the cause of the regional health gap and to work out solutions. This study aimed to analyze the factors associated with HRQoL in the Korean population aged over 65 years by region. Integrated raw data from the 2017 Korea Community Health Survey (n = 57,317), a cross-sectional study, were utilized. Based on the reduced Social-Ecological Model, the dependent variable was HRQoL and individual, interpersonal, and community factors were independent variables. Data were analyzed by complex-samples descriptive methods, and complex-samples general linear model (CSGLM). In both regions, individual factors (gender, age, household income, education level, drinking, BMI, sleep duration, the number of chronic illness, economic status, subjective health status, and physical activity), interpersonal factors (neighbor and friend contact frequency, religious activity, fellowship activity), and community factors (satisfaction with safety level, public transportation, and medical service) were commonly associated with HRQoL of elderly people (p < .05). In urban areas, household type and charity activity were associated with HRQoL, in contrast, neighboring help was associated with HRQoL in rural areas (p < .05). Based on the results of this study, multi-dimensional efforts considering personal, social, and environmental factors are necessary to improve HRQoL of the elderly. Furthermore, it implies that efforts should be made to ensure health equity through social support, or improvement of community factors considering regional characteristics.

